# Rational Design and Application of Covalent Organic Frameworks for Solar Fuel Production

**DOI:** 10.3390/molecules26144181

**Published:** 2021-07-09

**Authors:** Priyanka Verma, Joshua J.M. Le Brocq, Robert Raja

**Affiliations:** School of Chemistry, University of Southampton, University Road, Highfield, Southampton SO17 1BJ, UK; jjlb1g14@soton.ac.uk

**Keywords:** covalent organic frameworks, photocatalysis, CO_2_ reduction and water splitting

## Abstract

Harnessing solar energy and converting it into renewable fuels by chemical processes, such as water splitting and carbon dioxide (CO_2_) reduction, is a highly promising yet challenging strategy to mitigate the effects arising from the global energy crisis and serious environmental concerns. In recent years, covalent organic framework (COF)-based materials have gained substantial research interest because of their diversified architecture, tunable composition, large surface area, and high thermal and chemical stability. Their tunable band structure and significant light absorption with higher charge separation efficiency of photoinduced carriers make them suitable candidates for photocatalytic applications in hydrogen (H_2_) generation, CO_2_ conversion, and various organic transformation reactions. In this article, we describe the recent progress in the topology design and synthesis method of COF-based nanomaterials by elucidating the structure-property correlations for photocatalytic hydrogen generation and CO_2_ reduction applications. The effect of using various kinds of 2D and 3D COFs and strategies to control the morphology and enhance the photocatalytic activity is also summarized. Finally, the key challenges and perspectives in the field are highlighted for the future development of highly efficient COF-based photocatalysts.

## 1. Introduction

**Figure 1:**
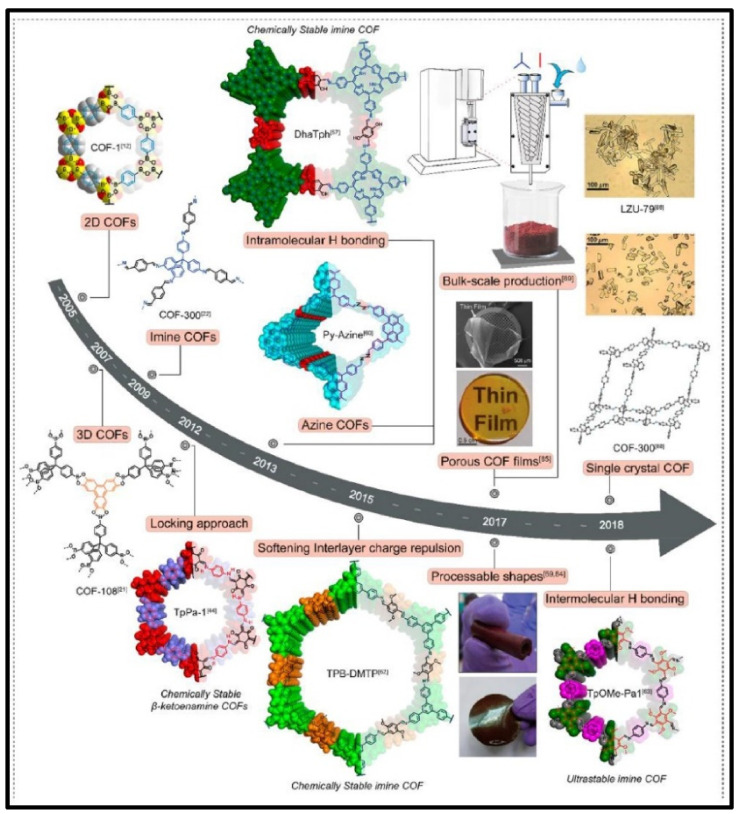
Synthesis strategies and timeline of development of COF materials. Reproduced with permission from reference [14]. Copyright 2021, Elsevier.

**Figure 2:**
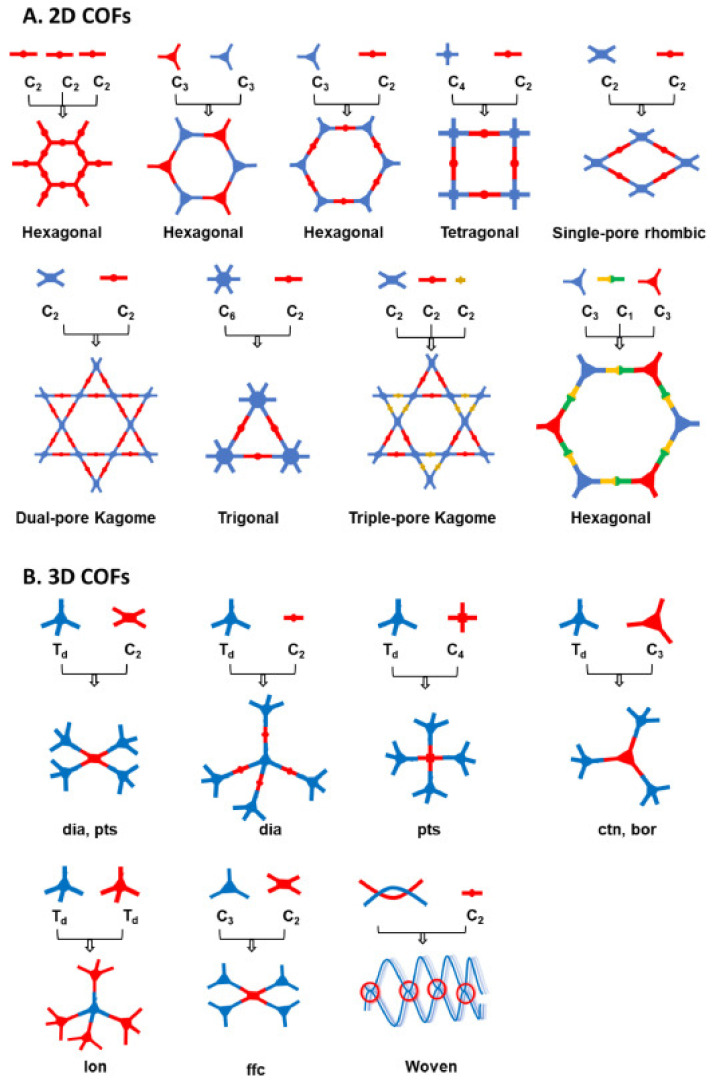
Schematic representation for design and construction of (**A**) 2D COFs and (**B**) 3D COFs. Reproduced with permission from reference [18]. Copyright 2021 Elsevier.

Covalent organic frameworks (COFs) are a new emerging class of porous and crystalline materials, which are made of organic groups linked together via robust covalent bonds [1,2,3]. The periodic arrangement of organic polymers is formed through imine, hydrazone, ketoenamine, and azine bond linkages. The first report on the synthesis of COFs by the condensation of boronic acid to form a boroxine-based framework was reported by Yaghi et al. in 2005 [4]. Since then, COFs have received significant attention and the rapid development in the design of crystalline porous materials has recently become an attractive field of research [5,6,7,8,9,10,11,12,13]. Figure 1 illustrates the timeline and several approaches for the synthesis of COF materials [14]. They can be constructed by using various building blocks to form two- or three-dimensional frameworks with structural tunability, following the reticular chemistry principle [10,12,13,15,16]. The two-dimensional (2D) COFs are formed by the stacking of atomic layers into overlapping layers through π–π interactions and extending these networks into three-dimensional arrangements results in 3D COFs [17]. Figure 2 displays the topology diagram to construct several types of 2D and 3D COFs with different linkers and knots, details of principles and strategies can be referred to in a recently reported review article [18]. The precise and predictable structures of COFs enable the understanding of their structure-property relationships [19,20]. They offer a platform for designing ordered organic structures, which are capable of displaying excellent performances in catalysis [21,22,23], gas storage [24,25], and ion conduction [26,27]. 

COFs display special characteristics when compared with traditional porous materials such as mesoporous silica [28,29], metal organic frameworks (MOFs) [30] and zeolites [31,32,33]. The tunable porous structure of COFs can display even superior reaction yields in comparison to the other semiconductor based photocatalysts such as graphitic carbon nitride (g-C_3_N_4_) [34,35]. In 2008, Jiang et al. reported the semiconducting behavior of boronic ester-based terephthalaldehyde (Tp)-COF along with the photocurrent measurements in the self-condensation of ppy-COF (ppy; pyrenediboronic acid) [36]. It has been reported that the pore wall can serve as reaction sites for the faster immigration of charge carriers under light irradiation conditions. The advantages of using COFs as photocatalysts materials are; (i) easy tunability of band structure and morphology by incorporating various building blocks; (ii) higher surface area and porous structure creating more active sites and providing easy accessibility of the substrate molecules when compared with traditional silica and zeolite materials; (iii) higher thermal and chemical stability due to the covalent bond linkages unlike coordinate bonds in MOFs; (iv) strong π–π interactions between the layers assisting in the charge carrier transportation; and (v) the appropriate bandgap enables the visible-light responsive ability. Further, the bandgap engineering can be tailored by the choice of suitable monomers and diverse functionalities. Covalent triazine based frameworks (CTFs) are another class of porous polymers made of aromatic triazine linkages containing several nitrogen functionalities [37,38,39,40]. The presence of triazine units within the framework allows the stabilization of heterostructures via strong metal-nitrogen interactions, resulting in a stable, photo-responsive framework when compared to the first type of COFs (COF 1 and COF 5), which were unstable in aqueous media. 

In recent years, the number of publications exploring the photocatalytic applications of COFs in hydrogen generation and CO_2_ reduction has recently seen a sharp increase. Therefore, a review article that focuses on more recent studies and applications is highly desirable, and will benefit the researchers interested in this exciting field. Other than hydrogen evolution and CO_2_ reduction, COFs have also been employed in several other reactions. For example, Yang et al. studied the visible-light-driven aerobic oxidation of small organic molecules using 2D-COF-1 under mild reaction conditions. The molecular oxygen activation by COF-based photocatalyst displayed high efficiency and good functional group tolerance [41]. A new COF material decorated with Pt was reported to be photoactive in the decarboxylative difluoroalkylation and oxidative cyclization reaction [42]. Several other reports explored the photocatalytic degradation of organic pollutants, dyes, chlorinated biphenyls [43,44]. A mechanochemical approach of ball milling synthesis method for TpMA (1,3,5-triformylphloroglucinol melamine)-COF material was explored in the photocatalytic degradation of phenol [45]. A two-component triazine based COF with Ni-single sites was designed for the first time in the sulfur-carbon cross-coupling reaction [46].

This article highlights recent developments in synthetic methodologies of COF frameworks for their application in the photocatalytic hydrogen evolution and CO_2_ utilization reactions, with a view to mapping structure–activity relationships. The synthesis protocols, novelty aspect, and the photocatalytic performances has been summarized along with a brief discussion of their mechanistic pathways under visible light irradiation conditions. Finally, we conclude by presenting our views on the current challenges and future directions in the strategic design and applications of COF-based hybrid photocatalysts.

## 2. COFs-Based Hybrids for Photocatalytic H_2_ Generation

Photocatalytic hydrogen evolution from water is a promising and sustainable approach to convert solar energy into chemical energy. Hydrogen is a clean fuel and can be produced by using renewable sunlight and water or hydrogen storage materials in the presence of a suitable photocatalyst [47]. Recently reported studies have revealed the affinity of COFs for generating hydrogen from water because of their ordered and crystalline structure [48,49,50,51,52]. In this section, we will discuss the recent reports investigating photocatalytic hydrogen generation using COF-based hybrid catalysts. Table 1 and Table 2 enlist the synthesis and novelty aspect of the reported COF-hybrid frameworks along with a brief comparison of their photocatalytic performance in the hydrogen generation reaction. 

**Figure 3:**
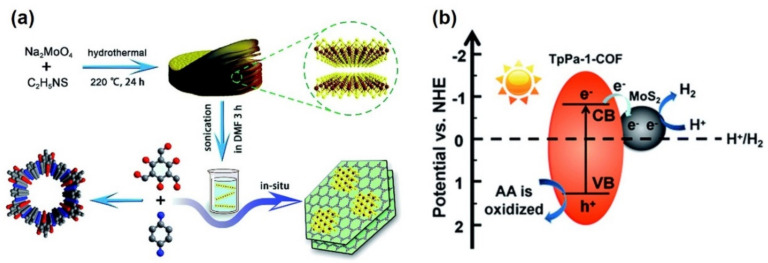
(**a**) Schematic illustration of the synthesis procedure of MoS_2_/TpPa-1-COF composite material and (**b**) mechanistic illustration of photocatalytic hydrogen evolution using MoS_2_/TpPa-1-COF under visible light irradiation. Adapted with permission from reference [53]. Copyright 2019, The Royal Society of Chemistry.

In general, COF based photocatalysts are used in conjunction with noble metal nanoparticles (NPs) as a cocatalysts to reduce the recombination rate of photogenerated charge carriers. Zhang et al. reported the ketoenamine-based covalent organic framework combination with non-noble metal MoS_2_ for the first time in 2019 in order to find an alternative to scarce and expensive noble metal NPs [53]. An exfoliated MoS_2_ dispersion of *N,N*-dimethylformamide (DMF) was used for the in situ growth of TpPa-1-COF to form a MoS_2_/TpPa-1-COF composite material. DMF was used as the solvent in order to exfoliate MoS_2_ via sonication to form ultrathin nanosheets, as shown in Figure 3a. The SEM images display a flower-like nanorod morphology of TpPa-1-COF and a distribution of MoS_2_ nanosheets over TpPa-1-COF was observed for MoS_2_/TpPa-1-COF. The optical absorption intensity of the composite was observed within the range of 200–800 nm, which was much more intense than the individual components. The valence band (VB) and conduction band (CB) calculation of the MoS_2_/TpPa-1-COF composite was estimated to be more negative than H^+^/H_2_ and more positive than O_2_/H_2_O potential, respectively, hence confirming its ability for water splitting.

With an optimum loading of 3 wt % MoS_2_, the nanocomposite produced hydrogen with an evolution rate of 55.85 µmolh^−1^ under visible light irradiation with an apparent quantum efficiency of 0.76% at 420 nm. The obtained catalytic performances were found to be better than pure TpPa-1-COF (1.72 µmolh^−1^) and Pt/TpPa-1-COF (54.79 µmolh^−1^). The composite displayed excellent stability for five recycling tests without any decrease in the photocatalytic activity. The mechanistic pathway revealed the role of MoS_2_ in facilitating the transfer of photogenerated electrons from COF to MOS_2_, thereby reducing the recombination rate of charge carriers, as studied by photoluminescence, time-resolved fluorescence, and electron paramagnetic resonance spectroscopy. The electrons accumulated on MoS_2_ act as a reductant, producing H_2_, and the sacrificial agent, ascorbic acid as illustrated in Figure 3b, consumes the corresponding holes created in the VB. This work can be cited as one of the first examples in combining the non-noble metal catalysts with COFs for enhanced hydrogen evolution activity.

**Figure 4:**
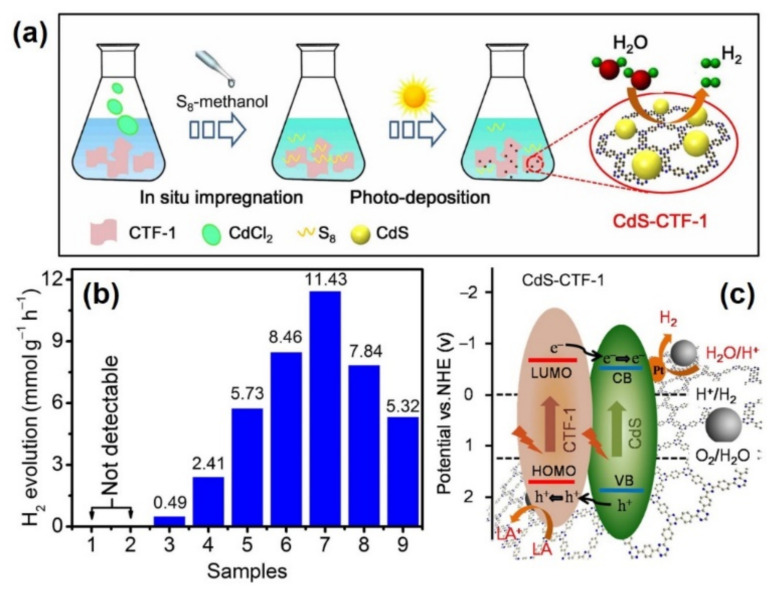
(**a**) Schematic illustration of the formation of CdS-CTF-1 nanocomposite, (**b**) hydrogen evolution reaction over (1) no catalyst, (2) without light, (3) CTF-1, (4) CdS, (5) 5%CdS-CTF-1, (6) 10%CdS-CTF-1, (7) 20%CdS-CTF-1, (8) 40%CdS-CTF-1 and (9) 20%CdS/CTF-1, (**c**) proposed mechanism for hydrogen generation over CdS-CTF-1. Adapted with permission from reference [54]. Copyright 2020, Elsevier.

The suitable band gap of CdS (2.4 eV) and its size dependent electronic properties makes it an ideal semiconductor photocatalyst for solar light utilization. Very recently, Wang et al. pioneered the approach of combining CdS NPs with covalent triazine based frameworks (CTFs) to form CdS-CTF-1, via a method combining photodeposition and impregnation. In this method, a suspension of CTF-1 and CdCl_2_ in absolute methanol was prepared, and S_8_ was subsequently added dropwise under an inert atmosphere with visible light irradiation. The conduction band electrons formed by CTF-1 upon light irradiation assist in the reduction of Cd^2+^ to Cd to form CdS NPs as shown in Figure 4a. SEM images revealed the formation of much smaller CdS NPs when combined with CTF-1 indicating the importance of CTF-1 layers to achieve highly dispersed NPs. For comparison of the catalytic activities, CdS/CTF-1 nanocomposite was also prepared by a solvothermal synthesis method. The interaction between CdS and CTF-1 to form a heterojunction was studied by the observed shift in the binding energy values in the Cd, S, and N XPS spectra. The photocatalytic performance for hydrogen generation was tested in the presence of lactic acid (LA) as a sacrificial agent and Pt as a cocatalyst over different samples as shown in Figure 4b. The superior catalytic performance with an optimum 20% CdS in CdS-CTF-1 was ascribed to the smaller sized CdS NPs and close contact between CdS and CTF-1 layers assisting in enhancing the separation efficiency of charge carriers. The charge carriers are formed by both CdS and CTF-1 under visible light irradiation conditions as illustrated in Figure 4c. As a result, the catalytic cycle for reducing water to H_2_ takes place through a transfer of electron from CTF-1 to CdS, and then CdS to the Pt NPs. Holes migrate from CdS to CTF-1, where they are quenched by the sacrificial agent, LA.

The special affinity of Au towards the thioether functional group was studied by functionalizing a COF with a thioether group (TTR-COF), which assisted in the specific adsorption of Au over other alkaline and alkaline-earth metal cations in seawater. This was then used to produce hydrogen under visible light irradiation conditions [55]. The 2D thioether functionalized COF was synthesized by a condensation of 1,3,5-tris(4-formylphenyl)triazine (TFPT) and 2,5-bis(2-(ethylthio)ethoxy)terephthalohydrazide (BETH) under solvothermal conditions to form TTR-COF. The Fourier transform infrared (FT-IR) spectra confirmed the successful formation of the COF by the appearance of a hydrazone bond peak at 1610 cm^−1^ and the disappearance of the aldehyde and amine group peak from TFPT and BETH, respectively. The photocatalytic H_2_ evolution from seawater displayed a reaction rate of 141 µmolh^−1^g^−1^ with a stable activity for up to five catalytic cycles. An analogous COF without thioether functional group was prepared (TFPT-COF) to compare H_2_ evolution activities. A significantly superior reaction rate for TFPT-COF was observed, but it decreased significantly with subsequent runs unlike TTR-COF, with a stable performance lasting up until five catalytic cycles. The transmission electron microscopy (TEM) image of the recovered catalyst in TFPT-COF displayed aggregated Au NPs, whereas TTR-COF displayed a uniform dispersion of Au NPs with an average particle size of 3.4 nm. The affinity of Au towards thioether stabilizes the Au NPs in TTR-COF, resulting in a higher catalytic stability. The conjugated framework of TTR-COF facilitates the transportation of charge carriers in which the photogenerated electrons migrate to the Au cocatalyst to carry out the H_2_ generation from water.

Zhang et al. reported a Knoevenagel condensation approach to prepare a 2D triazine-based COF with unsubstituted olefin linkages [56]. The two different monomers of aldehyde i.e., linear and trigonal, were used to prepare different COFs named as g-C_18_N_3_-COF and g-C_33_N_3_-COF, respectively. The frameworks displayed strong absorption in the visible region with a peak maximum at 400–450 nm, suggesting their efficient light harvesting ability. The photocatalytic hydrogen evolution was carried out in the presence of ascorbic acid (1 M) as a sacrificial agent and 3 wt % Pt as a cocatalyst under visible light irradiation. The significantly superior catalytic activity of g-C_18_N_3_-COF (14.6 µmolh^−1^) over g-C_33_N_3_-COF (3.7 µmolh^−1^) was ascribed to their intrinsic structural properties. The photoluminescence (PL) emission spectra at an excitation wavelength of 365 nm was red shifted by ca. 25 nm for g-C_18_N_3_-COF in comparison to g-C_33_N_3_-COF. This shift indicates the larger π-extended structure of g-C_18_N_3_-COF with a much longer lifetime of photogenerated excitons of 7.25 ns indicating the decreased rate of charge recombination and hence superior photocatalytic activity. The apparent quantum yield (AQE) was found out to be 1.06% under 420 nm monochromatic light irradiation. This work can be cited as one of the early reported examples for the synthesis of 2D COFs with enhanced stability and extended backbones displaying superior hydrogen evolution under visible light irradiation.

**Figure 5:**
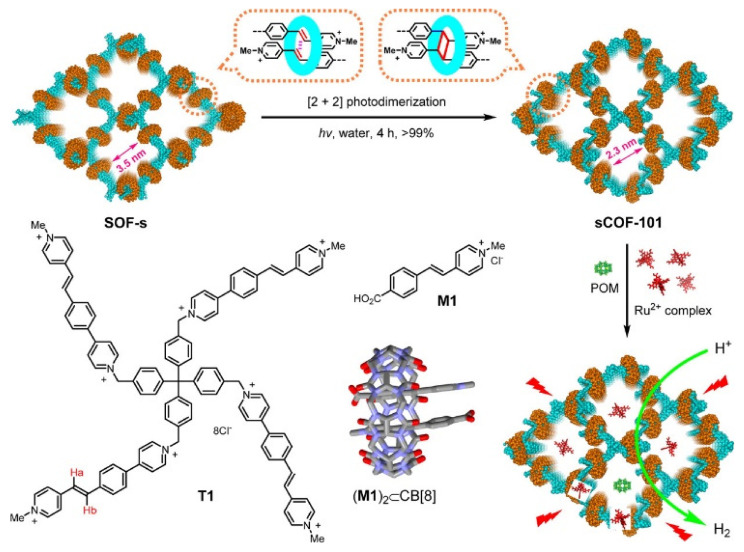
Monomers T1 and M1, the crystal structure of complex (M1)_2_ ⊂ CB [8] (CCDC no. 1951214) and schematic representation of SOF-s, water-soluble covalent organic framework sCOF-101 and its promotion of visible light-induced reduction of protons into H_2_ through enrichment of POM catalysts and Ru^2+^-complex photosensitizers. Reproduced with permission from reference [57]. Copyright 2020, American Chemical Society.

Most of the COFs currently available are insoluble in water or decompose upon dissolution, restricting their applications. Li et al. reported the synthesis of the first water-soluble 3D COF, named sCOF-101 by using a supramolecular organic framework (SOF) as a template for the [2+2] photopolymerization reaction between styryl pyridinium units [57]. The SOF was found to be stable at room temperature, but the T1 molecules underwent photodimerization with cucurbit [8] uril (CB [8]) under visible light irradiation to form s-COF-1 as shown in Figure 5. In the absence of CB [8], the photodimerization of T1 afforded the formation of an irregular porous polymer, depicted as P-irr. The evaluation of photocatalytic efficiencies of s-COF-101 was carried out in the presence of a ruthenium complex, used as a photosensitizer, and redox active polyoxometalate (POM) under visible light irradiation conditions. The s-COF-101 displayed TON almost 4 times higher than P-irr mediated catalysis. It was speculated that the presence of s-COF-101 enhances the electron transfer from photoexcited Ru^2+^ to the POM species, enhancing hydrogen evolution whilst irradiated with visible light.

**Figure 6:**
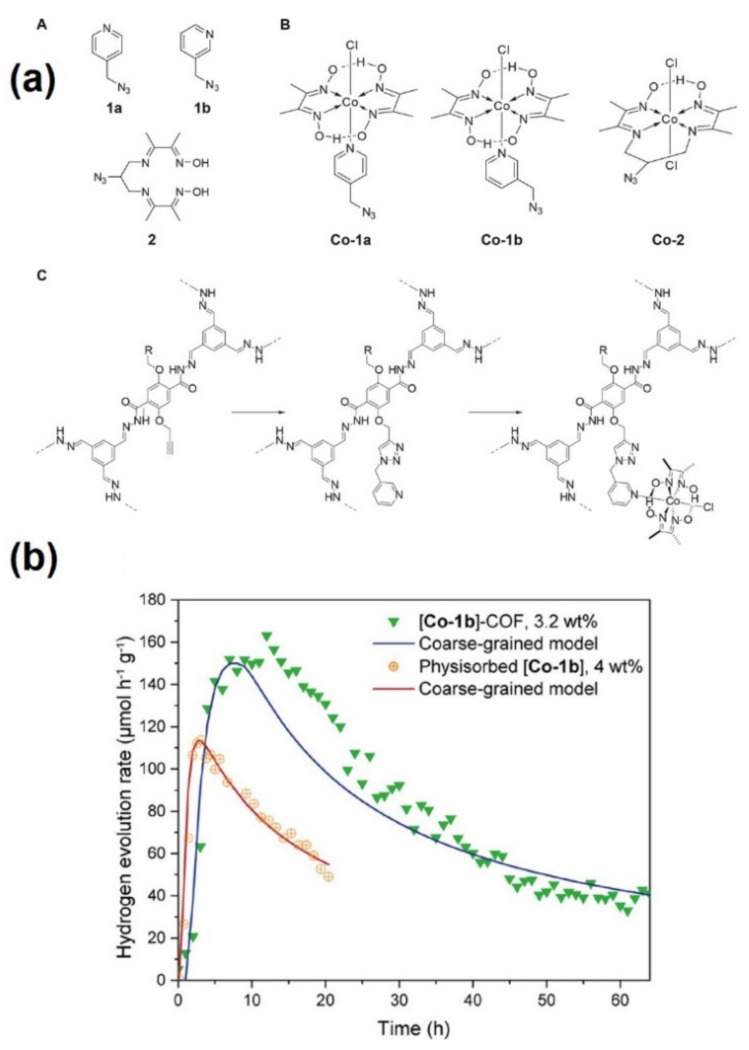
(**a**) (A) Structure of the azide-functionalized ligands 1a, 1b, and 2 and (B) the azide-functionalized complexes Co-1a, Co-1b, and Co- 2. (C) Post synthetic COF modification toward [Co-1b]−COF, (**b**) projection of the hydrogen evolution of [Co-1b]−COF containing 3.2 wt % [Co-1b] and COF-42 with 4.0 wt % physisorbed [Co-1b] based on the coarse-grained models. Adapted with permission from reference [59]. Copyright 2020, American Chemical Society.

The first report on the synthesis and characterization of crystalline mesoporous COF-42 framework was reported by Yaghi’s group in 2011 [62]. The condensation of hydrazine and aldehyde groups to form hydrazone-linked structure was controlled by the pH dependence of the hydrazone linkages. The solvothermal acid catalyzed condensation between 1,3,5-triformylbenzene (TFB) and 2,5-diethoxyterephthalohydrazide (DETH) in mixtures of mesitylene, 1,4-dioxane and aqueous acetic acid was carried out to prepare a pale-yellow microcrystalline powder of COF-42. The formation of new C=N bond by the condensation reaction was confirmed by appearance of a resonance signal at 149 ppm in the ^13^C CP-MAS spectrum. Further, the FT-IR spectra displayed stretching modes of ν_C_=_N_ and ν_C_=_O_ at 1621–1605, 1226–1203, and 1659 cm^−1^, suggesting the formation of the extended COF network. The pioneering investigation of this COF framework for photocatalytic hydrogen evolution was carried out much later in 2017 by the Lotsch group [58]. Using an azine-linked N2-COF with chloro(pyridine)cobaloxime as a cocatalyst and TEOA as an electron donor, a reaction rate of 782 µmolh^−1^g^−1^ was obtained. Very recently, the same group has reported the immobilization of azide-functionalized chloro(pyridine)cobaloxime on a COF-42 backbone and compared their photocatalytic activities with respect to the physisorbed systems [59]. The covalent attachment of the cobaloxime cocatalyst was carried out by creating functional sites within the COF framework in which one of the starting materials, DETH, was replaced with propargyl-containing 2,5-bis(prop-2-yn-1-yloxy)terephthalohydrazide (DPTH) to form modified pCOF_10_. The pCOF_10_ was functionalized by a click-chemistry approach as depicted in Figure 6a. The photocatalytic activities depicted an activity maximum instead of constant behavior as shown in Figure 6b. The cobaloxime covalently immobilized in the COF framework displayed significantly superior activities (47% more active) than the physisorbed samples. A superior hydrogen evolution rate of 163 µmolh^−1^g^−1^ for the hybrid sample compared to the physisorbed sample (111 µmolh^−1^g^−1^) was attributed to the close contact between the cobaloxime and COF pore wall as studied by solid-state NMR. This facilitated the charge transfer from COF to cobaloxime, as evidenced by the photoluminescence spectroscopic analysis. The interaction between both the components (cobaloxime and COF) leads to the improved photocatalytic activity and prolonged stable activity of the hybrid samples in comparison to the physisorbed variant.

**Figure 7:**
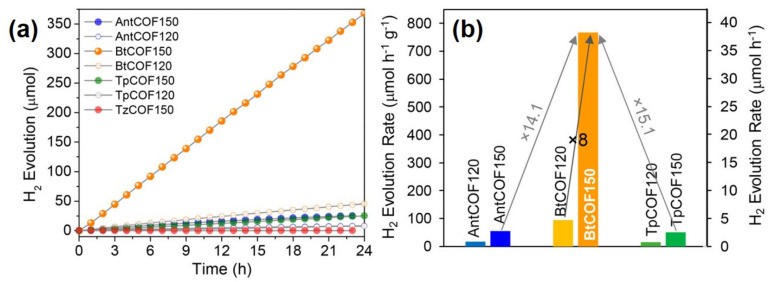
(**a**) Time course for photocatalytic H_2_ production under visible light (≥400 nm) of all of the prepared COFs (20 mg COF, 1 wt % Pt, water−TEOA (4:1, 100 mL, pH 11). (**b**) Comparison of photocatalytic hydrogen evolution rates. Adapted with permission from reference [60]. Copyright 2020, American Chemical Society.

Ghosh et al. reported a series of isoreticular COF frameworks in which TpCOF was prepared by the condensation of 4,4″-diamino-substituted *p*-terphenyl (Tp) and 1,3,5- triformylphloroglucinol (TH) in mesitylene, dioxane and aqueous acetic acid mixture at 120 and 150 °C. The isoreticular COFs were synthesized by substituting the central benzene ring of Tp with anthracene (Ant), benzothiazole (Bt), and tetrazine (Tz) to form AntCOF, BtCOF and TzCOF, respectively. As shown in Figure 7a,b, BtCOF150 displayed a maximum hydrogen evolution rate of 750 µmolg^−1^h^−1^, in the presence of 1 wt % Pt as a cocatalyst, using triethanolamine as the sacrificial electron donor. This activity was significantly greater than all other COFs tested in the series. Interestingly, authors have concluded the significance of light absorption and charge carrier generation in influencing the photocatalytic hydrogen evolution compared to other factors, such as surface area, crystallinity, layer stacking, and morphology of COF frameworks.

Zhang et al. also investigated the molecular engineering of COF frameworks by varying the ratio of ketoenamine and imine moieties to alter the structure, band gap, and light harvesting efficiency [61]. The covalent integration of COFs with MXenes (ATNT hybrid) displayed photocatalytic hydrogen evolution 12.6 times higher than the pristine COF with a reaction rate of 14,228.1 µmolg^−1^h^−1^. This improvement in the catalytic activity was attributed to the creation of a heterojunction, leading to an efficient charge transfer pathway due to the synergistic effect of photoactive COF and conductive MXenes. An apparent quantum efficiency (AQE) of 9.75% was reported for the ATNT hybrid exceeding the activities reported in the literature so far, for other COF-based photocatalysts. This hybrid COF material was found to be the most active catalyst for the hydrogen evolution reaction based on the reaction rate and quantum efficiency values. 

The structure–activity correlations based on the topology and design of active sites to maximize the photocatalytic activity in the hydrogen generation is summarized in Table 3. Amongst various hybrid frameworks, ATNT hybrid-based COF represented the highest photocatalytic activity (14,228 µmolh^−1^g^−1^). This included the use of MXene as the electron transfer mediator along with Pt cocatalyst as the reductant. The arrangement of 2D parallel layers of COF facilitated the conjugation effect for faster reaction rates. CdS-CTF-1 was another active COF framework where CdS and CTF-1 played an important role in the light absorption and Pt was used the cocatalyst reductant. The small sized CdS NPs created larger number of active sites leading to superior photocatalytic activity. The least active catalyst (141 µmolh^−1^g^−1^) was a thioether functionalized TTR-COF, which helped in the selective adsorption of Au ions form the seawater to facilitate the hydrogen generation reaction. The other reported photocatalytic systems for hydrogen generation (Table 4) are 1.0 wt % Au-Ag/TiO_2_ (12,820 µmolg^−1^h^−1^) [63], Ni-Cd/CdS (11,570 µmolg^−1^h^−1^) [64], FeCu(1:1)@C/g-C_3_N_4_ (722 µmolg^−1^h^−1^) [65], Ni_2_P/ZnIn_2_S_4_ (2066 µmolg^−1^h^−1^) [66], Cu-nanodiamond (1597 µmolg^−1^h^−1^) [67], and C_3_N_4_-MoS_2_ nanocomposite (12,778 µmolg^−1^h^−1^) [68].

## 3. COFs-Based Hybrids for Photocatalytic CO_2_ Conversion

CO_2_ reduction is widely regarded as a technology essential for reducing emissions and providing an alternative means to fossil fuel-based feedstocks, allowing for the sustainable growth and development of human society, using renewable solar energy. An ideal catalyst for CO_2_ should demonstrate a stable chemical structure, with a band gap suitable for efficiently harvesting visible light, an efficient electron transport, while being strongly absorbing and highly activating. To this end, covalent organic frameworks (COFs) prove an ideal candidate. They represent a class of crystalline polymers, which consist of strong covalently bonded, and highly conjugated bonding systems, with a repeating lattice structure resulting in ordered micropores. The conjugated system promotes an excellent electron mobility, and the covalently bonded system promotes a high thermal and photo-stability. To address the shortfalls of these materials, such as a rapid electron/hole recombination, a variety of inorganic dopants may be introduced, through general wetness impregnation or into vacant coordination sites included in the COF periodic structure. These dopants can serve as active sites, enhancing photocatalytic activity, as well as uptake. Table 5 serves as a summary for synthetic conditions, with Table 6 demonstrating catalytic activity, from the most active to the least active of discussed examples.

**Figure 8:**
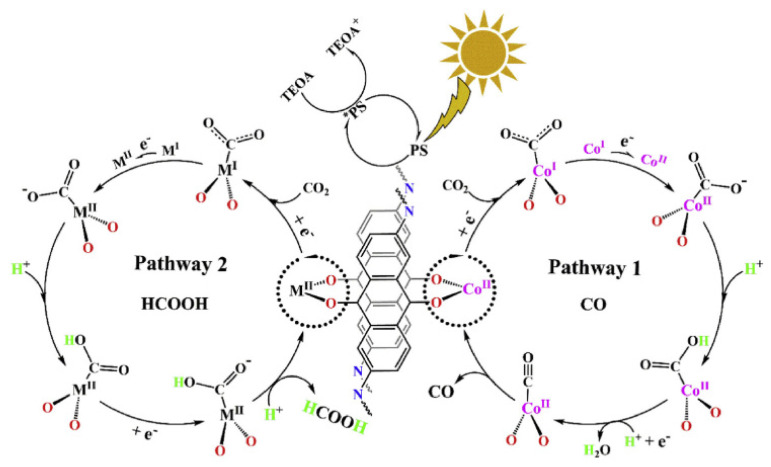
Proposed CO_2_ reduction mechanism over DQTP- TMI COF, highlighting pathways to CO and HCOOH. Reproduced with permission from reference [77]. Copyright 2019, Elsevier.

Metal dopant choice was found to strongly influence CO_2_ reduction pathways, and the subsequent catalytic activity, in conjunction with the band gap choice of the COF, and the experimental conditions, as highlighted in Figure 8 [77].

Formic acid has been thought to be a suitable hydrogen storage medium, with the ability to decompose to CO_2_ and H_2_, with a high volumetric capacity (53 g H_2_ L^−1^), and low toxicity and flammability under ambient conditions [78]. CO, CH_3_OH, and CH_4_ also form important chemical feedstocks for assembly into larger hydrocarbons. CO is part of syngas, which can be utilized in the Fischer–Tropsch reaction to form longer hydrocarbons, for use in carbon-neutral fuels [79]. CH_3_OH can be converted into olefins, via the methanol-to-olefin process (MTO), forming the basis for basic chemical feedstocks, or polymer manufacture [80]. Methane is already widely utilized as natural gas and may be burned in natural gas plants to generate energy or can be converted to syngas for longer chain hydrocarbon synthesis. 

The use of a photosensitizer and a sacrificial electron donor have been found essential for effective photocatalysis. In the work of Fu et al., they demonstrate that undoped ACOF-1 and a triazine framework (based on 2,4,6-tris(4-bromophenyl)-1,3,5-triazine) yield a low activity when tested at 4 atm of CO_2_, at 80 °C, with 8.6 μmolg^−1^ of MeOH for ACOF-1, and 13.7 μmolg^−1^ for N_3_-COF [71]. When compared to other materials examined in Table 6, which utilize metal dopants, photosensitizers, and sacrificial electron donors, they demonstrate the lowest activity. However, a key difference is that the N3-COF demonstrates a higher activity than ACOF-1, which instead consists of an azine linked benzene structure. Triazine-based COFs tend to give a higher performance than non-triazine systems as they have a higher affinity for negative charge when conducting electrons [69,70,71,81]. However, one shortfall of organic semiconductors is a rapid electron-hole recombination [74]. A photosensitizer prevents this, facilitating a higher catalyst efficiency, and may be used as a homogeneous co-catalyst, or itself be incorporated into the COF structure post-synthetically.

**Figure 9:**
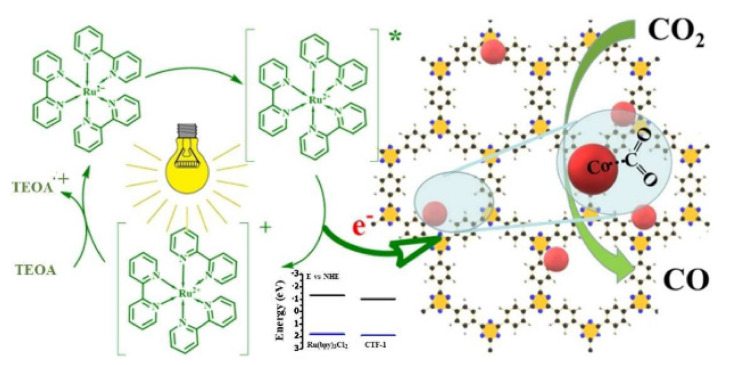
Interaction of a [Ru(bpy)_3_]^2+^ with sacrificial donor TEOA and Co-CTF framework. Reproduced with permission from reference [69]. Copyright 2019, Wiley-VCH.

For example, Bi et al. report that the inclusion of Co^2+^ into a triazine based CTF-1 causes a 44-fold enhancement (48 μmolg^−1^ CO) in catalytic activity over that of the pristine framework (1.1 μmolg^−1^ CO) [69]. Notably, when comparing to the work of Fu et al., both a photosensitizer, [Ru(bpy)_3_]^2+^, and a sacrificial donor, triethanolamine (TEOA) are used. As Figure 9 outlines, the photosensitizer is excited by incoming light, and separates the electron/hole pair by transferring the generated electron to the COF, which reduces CO_2_. TEOA then sacrifices an electron, quenching the photosensitizer. Cobalt was thought to enhance this mechanism through providing single atom active sites, which can readily adsorb CO_2_, as well as restraining electron/hole recombination rate within the COF. CO_2_ adsorption was found to be higher for the impregnated Co-CTF (9.76 cmg^−1^) compared to the pristine (7.97 cmg^−1^) and normalized to surface area. Zeta potential findings indicated that Co provides suitable Lewis acid sites for the coordination of CO_2_. Photoluminescence spectroscopy found that the Co-CTF demonstrated a longer lifetime than that of the pristine, demonstrating the longer-lived charge carriers in the Co-CTF.

Researchers have further experimented with metal dopant inclusion into COF structures by integrating the photosensitizer as part of a COF structure. For example, the work of Fu et al. demonstrates a 2D sp^2^c bipyridyl based COF, synthesized via a Knoevenagel condensation, in which a photosensitizer like-complex of Re(bpy)(CO)_2_Cl has been incorporated into the structure of the framework, through the bipyridyl nitrogen binding sites [11]. Successful chelation was confirmed via XPS, where the Re doped structure gave a similar XPS trace as free [Re(bpy)(CO)_3_Cl], demonstrating the chelation of Re. This was also supported by FTIR observations, where in addition to the triazine vibration at 2217 cm^−1^, CO stretching bands of 1900, 1917, and 2024 cm^−1^ was observed. By including Re in the structure, a CO yield of 1040 μmolg^−1^ was obtained, whereas without Re, only traces of CO was observed, demonstrating the need for a photosensitizer to facilitate electron/hole generation. The interaction of Re with the framework was rationalized through the following observations. UV-Vis indicated a red shifting of the absorption edge of the material from 589 nm to 694 nm, demonstrating a slight alteration of bandgap on Re addition. With the addition of Re, the BET surface area of the pristine, 432 m^2^g^−1^ is lowered to 323 m^2^g^−1^, although the CO_2_ sorption capability of the material is improved, with the Re-COF adsorbing 1.7 mmolg^−1^ of CO_2_ at 273 K, with a high isosteric heat of adsorption, 31 kJmol^−1^. The photocurrent response of the COF is increased with the inclusion of Re, indicating a higher current density being generated, and the Nyquist plots of the Re-doped COF show smaller arc radii than the undoped material, signifying that the addition of Re aids in charge separation and transfer.

Guo et al. demonstrate that this effect is related to the percentage loading of metal, when doping ruthenium NPs into TpPa-1, where the 0 wt % loading demonstrated the lowest yield of CO at 32.4 μmolg^−1^h^−1^, with 1, 3, and 5 wt % yielding 41.8, 108.8, 61.8 μmolg^−1^h^−1^, respectively [73]. TpPa-1 was synthesized solvothermally under anaerobic conditions achieved through the evacuation and sealing of reactants in a glass ampoule. Ru was then introduced to the material via wetness impregnation, before being dried and reduced at 280 °C for 6 h. The known thermal stability of most COF structures at these temperatures permits elevated temperatures for post-synthetic treatment, although some evidence suggests that thermal treatment of 2D COFs can alter the periodic structure, which is not detectable via TGA [82]. The resulting Ru loading was determined via ICP-OES, with the nanoparticle morphology confirmed via HR-TEM, with [1 0 0] and [2 1 0] lattice planes evident, characteristic of metallic Ru. Notably, no Bragg peaks for Ru were observed via PXRD, indicating a high dispersal dispersion of Ru throughout the framework, also verified through EDX analysis. XPS analysis indicated a shift for the Ru 3p1/2 and 3p3/2 signals (484.3 and 462.1 eV, respectively) vs. the RuCl_3_ (485.6 and 463.4 eV) standard, indicating the complete reduction of Ru^3+^ to Ru^0^. Photoluminescence spectroscopy indicated that the shortest lifetime was observed with the 3 wt % loaded sample, and transient photocurrent responses indicated the highest charge density on irradiation with light. Both results suggest that 3 wt % is the optimal loading for charge separation. Nyquist plots also yielded the smallest arc radii for the 3 wt % sample. It was thought that by increasing the loading beyond 3 wt %, Ru clusters are being formed which begin to facilitate charge recombination, thus lowering the efficiency of the material.

Chen et al. demonstrated that the dopant species does not necessarily have to be a precious metal [70]. Their work focused on comparing polyimide linked COFs, with a [Ni(bpy)_3_]^2+^ photosensitizer. When comparing tris(4-aminophenyl)amine (TAPA), 1,3,5-tris(4-aminophenyl) benzene (TAPB), or 1,3,5-tris(4-aminophenyl)triazine (TAPT) structures connected via polyimide linkages, the azine-based COF gave the highest performance of 483 μmolg^−1^h^−1^, whereas only trace activity was observed for the other tested frameworks. The imide condensation was performed solvothermally, with the reaction mixture degassed via pump-thaw through flash freezing in liquid nitrogen, before being sealed and heated at 200 °C for 5 days. All materials indicated a successful condensation, with characteristic stretching bands of 1720–1725 cm^−1^ indicating the 5-member imide ring formation, and an imide carbonyl signal observed at 165 ppm via ^13^C NMR. All materials matched with their simulated PXRD patterns, with a weakening of the [1 1 0] peak on Ni addition, suggesting an increase of disorder for this lattice plane. The surface area values for the materials were within expected ranges, with 475, 1175, and 825 m^2^g^−1^ for TAPA, TAPB, and TAPT, respectively, with all materials exhibiting a pore size of 1.5–3.5 nm, modelled using nonlocal density functional theory (NLDFT). The TAPT catalyst demonstrated the highest isosteric heat of absorption adsorption of CO_2_ (29.76 kJ mol^−1^), indicating the attraction between the triazine functionality and CO_2_, and XPS data demonstrated that the doped [Ni(bpy)_3_]^2+^ was encapsulated in the pore system, based on an intensification of the Ni 2p signal through argon ion bombardment. When examined electrochemically, photocurrent density was found to be the highest for the TAPT material, mirroring catalytic results. To explore this further, EPR experiments were carried out, comparing a sample before and after irradiation. The shift in electron density to the triazine ring demonstrated the capability for the functional group to hold negative charge, facilitating charge separation compared to the other conjugated systems examined in this work.

**Figure 10:**
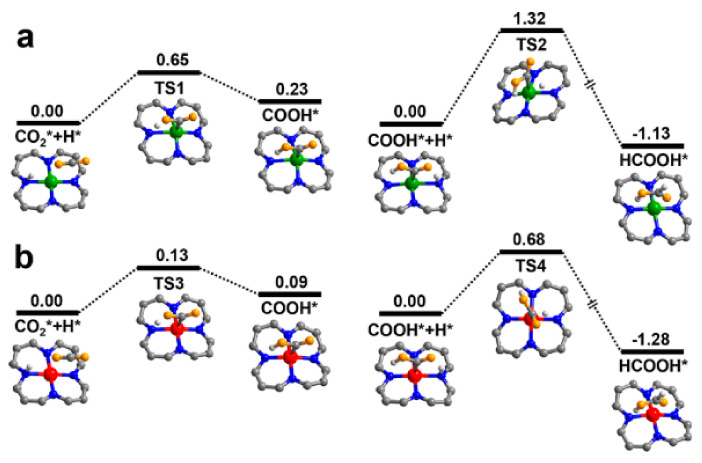
Calculated potential energy profile of CO_2_ to HCOOH, over (**a**) COF-367-Co^II^, and (**b**) COF-367-Co^III^. Co^II^, Co^III^, C, N, O, and H atoms are shown as green, red, gray, blue, orange, and light gray, respectively. Reproduced with permission from reference [72]. Copyright 2020, American Chemical Society.

The electronic environment of the dopant species was also found to be crucial for product selectivity, as found by Gong et al. [72]. When examining Co-COF-367, a porphyrinic COF, it was found that Co^II^-COF-367 has a product profile of 48.6, 16.5, and 12.8 μmolg^−1^h^−1^ of formic acid, CO and methane, whereas Co^III^-COF-367 yields 93.0, 0.44, and 10.1 μmolg^−1^h^−1^ of the respective products. Using ^13^C labelled CO_2_ as a feedstock, the researchers confirmed that all products are a result of photocatalytic reduction. EPR measurements indicated a strong signal for Co^II^ at g = 2.30 while the material was measured in the dark where the intensity decreased on irradiation with light. This was thought to signify the transformation of Co^II^ to Co^I^. The density functional theory (DFT)-based calculations indicate that the presence of Co^II^ is beneficial for forming formic acid, but prevents any further conversion, due to a high barrier energy for transformation into CO or CH_4_. Co^III^ dopants demonstrate a lower barrier energy, vindicating the experimental observations as shown in Figure 10. Photoelectrical experiments indicate that Co^III^-COF-367 has a higher charge separation efficiency than the Co^II^ structure, demonstrated by a longer photoluminescent activity with photoluminescence spectroscopy, as well as a higher photocurrent response.

**Figure 11:**
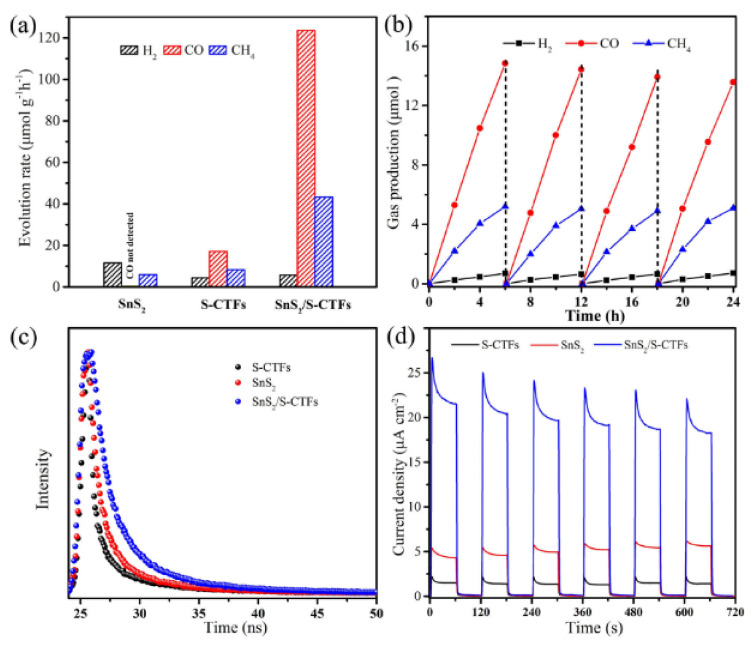
(**a**) Average gas production rates of SnS_2_, S-CTFs, and SnS_2_/S-CTFs. (**b**) Photostability of SnS_2_/S-CTFs for repeat CO_2_ reduction cycling. (**c**) Time resolved PL decay curves of S-CTFs, SnS_2_, and SnS_2_/S-CTFs. (**d**) Transient photocurrent responses under λ > 420 nm irradiation. Reproduced with permission from reference [74]. Copyright 2020, Wiley-VCH.

Guo et al. demonstrate that charge separation efficiency can be achieved through heterojunction structures, by doping a sulfur-bridged triazine COF with SnS_2_ [74]. The hybrid material demonstrated a greatly enhanced performance for CO_2_ photoreduction, demonstrating average evolution rates of CO and CH_4_ up to 123.6 and 43.4 μmol h^−1^g^−1^ respectively, when irradiated under visible (420 nm) light, as demonstrated in Figure 11a. By comparison, SnS_2_ evolved only CH_4_ at a rate of 5.9 μmol h^−1^g^−1^, whereas S-CTF evolved both CO and CH_4_ at rates of 17.1 and 8.3 μmol h^−1^g^−1^, respectively. The hybrid material also demonstrated significant photostability, with performance showing little attenuation after four 6-h cycles, shown in Figure 11b. By designing a Z-scheme heterojunction, catalysis shows that recombination of electron/hole pairs are hindered, increasing catalytic efficiency compared to the isolated materials. Photoluminescence spectroscopy demonstrated that recombination was significantly quenched for SnS_2_/S-CTF. The average PL lifetime demonstrated by the hybrid reached 7.18 ns, compared to 5.60 ns and 4.28 ns for SnS_2_ and S-CTF (Figure 11c). The S-CTF was synthesized solvothermally under anaerobic conditions via the nucleophilic substitution of cyanuric chloride and trithiocyanuric acid. SnS_2_ was doped in the form of NPs, which were formed in-situ by immersion of the S-CTFs in a SnCl_2_ ethanol solution at 180 °C. SEM imaging of the pristine S-CTF showed the formation of 400 nm smooth spheres. The introduction of SnCl_2_ triggered the formation of vertical platelets on the surface of the spheres, of which high resolution TEM indicates are composed of <5 nm nanocrystals. The lattice fringes of said crystals were found to correspond with the (1 0 1) and (1 0 0) planes of crystalline SnS_2_, supporting PXRD findings demonstrating diffraction planes of both S-CTF and SnS_2_ in the hybrid material. To further support the formation of a heterojunction, XPS was used to examine the nature of the binding energies of surface N species. Compared to the undoped S-CTF counterpart, the doped material exhibited an increase in N species binding energy by 0.5 eV. This was interpreted as evidence for an electron transfer from the framework to the inorganic nanoparticle. This resulted in a higher observed transient photocurrent response for the hybrid material (Figure 11d).

The morphology of COF nanostructures also plays an important role in affecting the photocatalytic performance. For the materials considered in this review in Table 5, all 2D structures have been found to have some of the highest performances. The 2D structures have more accessible pores than 3D bulk crystalline structures, as well as a better conductivity, which facilitates photoelectric efficiency [11,75,76,77]. As previously discussed, Fu et al. demonstrate a Re-functionalized 2D sp^2^c bipyridyl based COF, with a performance of 1040 μmolg^−1^h^−1^ [11]. The work by Li et al. demonstrates a similarly Re-functionalized 2D “TpBpy” COF, with a performance of 291 μmolg^−1^h^−1^ [75]. When compared to a homogeneous Re-bpy catalyst, the Re-COF gave a higher performance over time due to the porosity of the structure ensuring a higher contact time between the Re active site, compared to the exposure of the homogeneous catalysts which is based on CO_2_ diffusion through the reaction medium. According to CO_2_ sorption experiments, Re-TpBpy demonstrated an adsorption volume of 44 cm^3^g^−1^, whereas the adsorption volume was near 0 for the Re-Bpy catalyst. XPS validated the electronic interaction of the Re functionalization on the framework, with a shift of the pyridinic N 1s signal from 398.9 to 400.46 eV on Re addition. This was associated with a decrease of electron density as Re is complexed.

**Figure 12:**
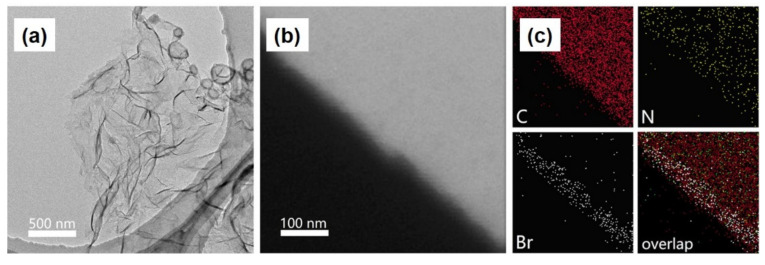
(**a**) TEM image (**b**) HAADF-STEM image and (**c**) elemental mapping of COF-367 nanosheets, modulated using 4-bromo-2,6- dimethyl benzaldehyde, with the lateral plane demonstrating a high concentration of the modulator. Adapted with permission from reference [76]. Copyright 2019, American Chemical Society.

Restricting morphology further to 2D nanosheets has been found to significantly impact the catalytic performance. Liu et al. demonstrate that by fabricating Co-COF-367 nanosheets using a modulated imine-exchange bottom-up synthesis strategy, catalytic activities of up to 10,162 and 2875 μmolg^−1^h^−1^ for CO and H_2_ respectively, were evidenced [76]. An analogous bulk Co-COF-367 demonstrated an activity of only 124 μmolg^−1^h^−1^. However, it is notable that in the absence of the [Ru(bpy)_3_]Cl_2_ photosensitizer or the ascorbic acid sacrificial agent, only trace activity was observed. Synthesis of the nanosheets used H_2_TAPP alongside 4,4′-biphenyldialdehyde (BPDA) as building units, with 2,4,6-trimethylbenzaldehyde (TBA) functioning as the modulator. By using 4-bromo-2,6-dimethylbenzaldehyde in place of the modulator, nanosheets were fabricated which bromine “marked” lateral planes, observable via elemental mapping, in Figure 12, which vindicated the postulated effect of the modulating agent. For purification, bulk COF impurities were separated from the final product through suspension and centrifugation at 2000 rpm, yielding a colloidal dispersion of the nanosheets. PXRD found that the nanosheets were not crystalline enough to yield meaningful diffraction peaks. As a result, scanning tunneling microscope (STM) was used to reveal the periodic pore lattice of the structure, giving lattice parameters of a = b = 2.7 nm, α = 90, which matched with the simulated structure. Sharp selected area electron diffraction (SAED) was used to highlight the ordered tetragonal structure of these sheets, to verify the lattice order of the sheets despite the low crystallinity. Bulk frameworks did yield a higher BET surface area, 467 m^2^g^−1^ of the bulk, vs. the 106 m^2^g^−1^ of the nanosheets.

Product modulation can also be achieved within 2D structures. Lu et al. demonstrated this with a 2D 2,6-diaminoanthraquinone-based COF, functionalized with Co, Ni and Zn [77]. The type of dopant-controlled CO production, with a 1002 μmolg^−1^h^−1^ production of CO with Co, and a 152.5 μmolg^−1^h^−1^ yield of formic acid over the Zn based material. The framework was synthesized via a Schiff-base condensation, and then metallated post-synthetically through refluxing with the metal salt in solution. XPS was used to verify the presence of metals, with the following environments found; Co 2p_3/2_ = 786.2 eV, as evidence for Co^II^, Ni 2p_3/2_ = 855.6 eV for Ni^II^, and Zn 2p_3/2_ = 1022.2 eV for Zn^II^. The Co energy is red shifted, which was taken as evidence for a Co-O bond with neighboring carbonyls, which donate electron density. Obvious bonding signals were not found for Ni and Zn dopants. Interaction with framework carbonyls was demonstrated further, through instead using a 2,6-diaminoanthracene group in place of the 2,6-diaminoanthraquinone. This demonstrated a significantly lower activity for Co dopants, but affected Ni and Zn dopants much less. These findings, amongst others, were used to elucidate a two-path mechanism for photoreduction over these systems, which was found influenced by metal type. In addition to this, Lu et al. also noted the importance of the sacrificial donor type. It was found that TEOA favors gaseous products CO, whereas TEA and TIPA favor formic acid. It is still a matter of discussion as to why this is the case.

Table 7 summarizes the structure-property relations of the catalytic systems discussed in this section for CO_2_ reduction reaction. The catalytic active sites and their topology has been correlated with their photocatalytic performance. The ultrathin imine-based Co-COF nanosheet framework with large surface area values displayed the most active photocatalytic reduction of CO_2_ to CO (10162 µmolh^−1^g^−1^). The Re-bpy-COF with bipyridine units exhibited second highest reaction rate due to the extended conjugation offered by the Re complex. According to the recently reported studies, the state-of-the-art photocatalysts for CO_2_ reduction also displays comparable activity to the COF materials [83,84]. Table 8 summarizes some of the photoactive materials including Ag-Cr/Ga_2_O_3_ (CO, 480 µmolg^−1^h^−1^) [85], Cu@V-TiO_2_/PU (CH_4_, 933 µmolg^−1^h^−1^) [86], ZnO nanosheets (CO, 406.77 µmolg^−1^h^−1^) [87], Rh-Au-SrTiO_3_ (CO, 369 µmolg^−1^h^−1^) [88], Mo-doped g-C_3_N_4_ (CO, 887 µmolg^−1^h^−1^) [89] and Nb-TiO_2_/g-C_3_N_4_ (CH_4_, 562 µmolg^−1^h^−1^) [90].

## 4. Conclusions and Perspectives

In this review, we have summarized some of the latest progress in the synthesis and photocatalytic application of COF hybrids in the hydrogen evolution and CO_2_ utilization reaction. The combination of COFs with CdS, MoS_2_, SnS_2_, Co, and Ru has been included, in which the COF-367-Co was found to be most active photocatalyst for CO_2_ reduction to CO with a reaction rate of 10,162 µmolg^−1^h^−1^ and ATNT hybrid COF displayed superior catalytic performance in hydrogen generation with a reaction rate of 14,228 µmolg^−1^h^−1^. From Table 3 and Table 7, summarized in the last section, it can be inferred that conjugation and porosity are the two of the main characteristics to achieve the superior photocatalytic performance in both the reactions. For the hydrogen generation reaction, the combination of COF with semiconductors such as MoS_2_ and CdS photocatalyst was one of the approaches to maximize the photocatalytic efficiency. However, the CO_2_ reduction reaction involves the use of redox active sites (such as Co), larger surface area values, and the presence of triazine/bipyridine units assisting in the chelation of metal complexes to facilitate the superior reaction rates. 

The use of COFs in photocatalysis provides the advantage of higher surface area, light harvesting ability, tunable porous structure, π-conjugation, and chemical stability [91]. In the last 15 years, COFs have emerged as a promising platform for the design and engineering of nanostructured catalysts. They have also gained significant research interest from interdisciplinary fields and therefore a standard nomenclature system for COFs is necessary at this stage in order to avoid publishing with different names for the same COF materials [92,93,94,95]. The research field is still in its infancy and several fundamental issues need to be addressed to establish practical applications. First, the preparation of COF materials is faced with many problems because of absence of standard synthesis conditions, for example, the type of reagents used, the process time and temperature. The preparation of monomers and reliable and reproducible large-scale COF synthetic techniques also need to be developed. The application of COFs owing to the presence of unique π-conjugated units requires further research and comparison with other porous counterpart materials like zeolites and mesoporous silica materials. Furthermore, more advanced specific characterization techniques are required to obtain detailed structural information of COFs and to further broaden the use of various different ligands other than the most commonly used porphyrin, imine and bipyridine [15,16]. 

Most reports are based on the use of noble metals and photosensitizers for hydrogen evolution and CO_2_ utilization reaction, which restricts their use in large-scale applications because of their impact on the cost of production [9]. The possible alternative to this could be the use of low-cost and earth abundant metals or designing novel single atom-based catalysts, which can be catalytically active, providing an economically viable solution. Their integration with plasmonic nanostructures can also be an interesting strategy to prepare novel photoactive materials where both the metal and support material is capable of light harvesting leading to enhanced reaction rates [96,97,98,99]. The effect of using different morphologies of COFs can be further explored to improve the photocatalytic efficiency of reactions [100]. We envisage that the field of COFs will further grow and attract researchers from the fields of physics, chemistry, materials science, and engineering to find solutions relating to energy and environmental issues [101,102,103].

## Figures and Tables

**Table 1:** Synthesis protocols and novelty aspects of hybrid COF-based frameworks explored in hydrogen evolution reaction.

COFs	Synthesis	Novelty	Ref.
MoS_2_/TpPa-1-COF	In situ growth of COF in an exfoliated MoS_2_ dispersion in DMF	MoS_2_ nanosheets evenly distributed on flower-like cluster TpPa-1-COF	[53]
CdS-CTF-1	Impregnation combined with photodeposition approach to disperse CdS NPs on triazine based COF	Smaller sized CdS NPs with more exposed active sites	[54]
TTR-COF	Condensation of 1,3,5-tris(4-formylphenyl)triazine (TFPT) and 2,5-bis(2-ethylthio)ethoxy)terephthalohydrazide (BETH) under solvothermal conditions	Selective adsorption of Au through affinity of thioether functionalized COF	[55]
(thioether functionalized)			
g-C_18_N_3_-COF and g-C_33_N_3_-COF	Knoevenagel condensation approach with linear and trigonal aldehyde monomers to form g-C_18_N_3_-COF and g-C_33_N_3_-COF, respectively	Honeycomb-like porous COF structures with sp^2^ carbon-linked triazine units	[56]
sCOF-101	An irreversible polymerization of supramolecular organic framework through cucurbit [8] uril [2+2] photodimerization	3D ordered porous COF with a channel diameter of 2.3 nm, capable of enriching photosensitizer and redox-active sites in water	[57]
N2-COF	Solvothermal acid catalyzed condensation between 1,3,5-triformylbenzene (TFB) and 2,5-diethoxyterephthalohydrazide (DETH)	An outer sphere of electron transfer from azine-linked N2-COF to cobaloxime co-catalyst, as revealed by quantum calculations	[58]
Cobaloxime immobilized COF-42	DETH was replaced with propargyl-containing 2,5-bis(prop-2-yn-1-yloxy)terephthalohydrazide (DPTH) to provide functional sites for covalent attachment of cobaloxime by click-chemistry approach	Propargyl-functionalized COF-42 with covalently tethered three different cobaloxime to form COF−cobaloxime hybrid catalyst	[59]
BtCOF-150	Substitution of the central benzene ring of TpCOF with benzothiazole (Bt) at 150 °C	The AA’ stacked BtCOF150 displayed improved charge migration	[60]
ATNT hybrid	Mixture of COF and NH_2_-Ti_3_C_2_T_x_ ultrasonicated followed by heating at 120° C for 3 days to form a dark red precipitate	The COF forms oriented 2D layers parallel to the substrate surface with vertically aligned π-columns in the ATNT hybrid	[61]

**Table 2:** COF-based hybrid materials for photocatalytic hydrogen generation reaction.

COFs	Type	Light Source	Reaction Conditions	Photocatalytic Performance (µmolh^−1^g^−1^)	Ref.
ATNT hybrid	Ketoenamine-based	300 W Xe lamp (λ > 420 nm)	Catalyst (10 mg), ascorbic acid (100 mg), 3 wt % Pt as cocatalyst	14,228	[61]
CdS-CTF-1	Covalent triazine-based	300 W Xe (λ > 420 nm)	Catalyst (20 mg), lactic acid (8 mL), water (80 mL)	11,430	[54]
MoS_2_/TpPa-1-COF	Ketoenamine-based	300 W Xe (λ > 420 nm)	Catalyst (10 mg), ascorbic acid (100 mg), water (50 mL)	5585	[53]
N2-COF	Hydrazone-based	300 W Xe lamp (I = 100 mW cm^−2^)	Catalyst (5 mg), triethanolamine (100 µL), acetonitrile (10 mL), chloro(pyridine)cobaloxime (400 µL, 2.48 mM) as co-catalyst	782	[58]
BtCOF-150 (Bt: benzothiadiazole)	Ketoenamine-based	300 W Xe lamp (λ > 400 nm)	Catalyst (20 mg), 1 wt % Pt, water−TEOA (4:1, 100 mL, pH 11)	750	[60]
g-C_18_N_3_-COF	2D triazine-based	300 W Xe (λ > 420 nm)	Catalyst (50 mg), ascorbic acid (100 mL, 1 M), 3 wt % Pt as cocatalyst	292	[56]
Cobaloxime immobilized COF-42	Hydrazone-based	300 W Xe lamp (I = 100 mW cm^−2^)	Catalyst (5 mg), acetonitrile (10 mL), triethanolamine (100 µL)	163	[59]
TTR-COF (thioether functionalized)	Covalent triazine-based	300 W Xe (λ > 420 nm)	Catalyst (20 mg), seawater (50 mL), triethanolamine (5 mL)	141	[55]
sCOF-101 (water soluble)	3D-supramolecular framework-based	300 W solid state light	Methanol (2 mL), pH = 1.8, [Ru(bpy)_3_]^2+^, POM	TON = 400	[57]

**Table 3:** Structure–activity correlations for enhancing the photocatalytic performance in the hydrogen generation.

COFs	Active Sites	Topology	Photocatalytic Performance (µmolh^−1^g^−1^)	Ref.
MoS_2_/TpPa-1-COF	TpPa-1-COF: light absorber	Nanoflower-like morphology of MoS_2_ sheets	5585	[53]
	MoS_2_: Reductant			
CdS-CTF-1	CTF-1, CdS: light absorber	Small sized CdS NPs leads to more active sites and superior activity	11,430	[54]
	Pt: cocatalyst, reductant			
TTR-COF	TTR COF: light absorber	Selective adsorption of Au through affinity of thioether functionalized COF	141	[55]
(thioether functionalized)	Plasmonic Au NPs: reductant			
g-C_18_N_3_-COF and g-C_33_N_3_-COF	Olefin linked COF: photosensitizer and active site	Honeycomb-like porous COF structures with sp^2^ carbon-linked triazine units	292	[56]
sCOF-101	COF: light absorber	3D ordered porous COF with a channel diameter of 2.3 nm, capable of enriching photosensitizer and redox-active sites in water	TON = 400	[57]
	Ru^2+^ complex: photosensitizer			
	Polyoxometalate: redox active sites			
N2-COF	COF: photosensitizer	An outer sphere of electron transfer from azine-linked N2-COF to cobaloxime co-catalyst, as revealed by quantum calculations	782	[58]
	Cobaloxime: co-catalyst (redox-active)			
Cobaloxime immobilized COF-42	COF: photosensitizer	Propargyl-functionalized COF-42 with covalently tethered three different cobaloxime to form COF−cobaloxime hybrid catalyst	163	[59]
	Cobaloxime: active site			
BtCOF-150	COF: photosensitizer	The AA’ stacked layers of BtCOF150 displayed improved charge migration	750	[60]
	Pt: cocatalyst (reductant)			
ATNT hybrid	COF: photosensitizer	The 2D parallel layers of COF to the surface with vertically aligned π columns in the ATNT hybrid	14,228	[61]
	MXene: electron transfer mediator			
	Pt: cocatalyst, reductant			

**Table 4:** State-of-the-art photocatalysts for hydrogen generation.

Photocatalyst	Light Source	Reaction Conditions	Photocatalytic Performance (µmolg^−1^h^−1^)	Ref.
Au-Ag/TiO_2_	250 W Hg lamp	Catalyst (14 mg), 20 vol % methanol in water	12,820	[63]
Ni-Cd/CdS	300 W Xe lamp (λ > 410 nm)	Catalyst (20 mg), Na_2_S (0.1 M), Na_2_SO_3_ (0.1 M)	11,570	[64]
FeCu(1:1)@C/g-C_3_N_4_	300 W Xe lamp	Catalyst (10 mg), 15 % TEOA in 100 mL water	722	[65]
Ni_2_P/ZnIn_2_S_4_	300 W Xe lamp (λ > 400 nm	Catalyst (50 mg), 10 vol % lactic acid in 100 mL water	2066	[66]
Cu-nanodiamond	300 W Xe lamp	Catalyst (100 mg), 20 vol % ethanol	1597	[67]
C_3_N_4_-MoS_2_ nanocomposite	400 W Xe lamp	Catalyst (3 mg), 20 vol % TEOA in 40 mL water	12,778	[68]

**Table 5:** Synthetic protocols and novelty aspects of hybrid COF-based frameworks explored for CO_2_ reduction.

COFs	Synthesis	Novelty	Ref.
Co-CTF-1	Aerobic acid catalyzed trimerization of terephthalonitrile, with wetness impregnation of Co	2D-layered COF porous structure with triazine units promoting CO_2_ adsorption and accommodation	[69]
[Ni(bipy)_3_]^2+^—PI-COF	Anaerobic solvothermal imide condensation between pyromellitic dianhydride and amine-terminated building units.	Hexagonal porous polyimide COF assisting in CO_2_ photoreduction	[70]
N_3_-COF, ACOF-1	Anaerobic solvothermal condensation between triformylbenzene and hydrazine hydrate (ACOF-1), and 2,4,6-tris(4-bromophenyl)-1,3,5-triazine with N-formylpiperidine (N_3_COF)	Coplanar azine and phenyl rings facilitating the conjugation effect	[71]
Re-bpy-sp^2^c-COF	Knoevenagel condensation of 1,3,6,8-tetrakis(4-formylphenyl)pyrene and 5,5′-bis(cyanomethyl)-2,2′-bipyridine. Post synthetic ligation of [Re(CO)_5_Cl].	Porous crystalline COF with bipyridine units ligated to Re complex, forming fully π-conjugated backbone	[11]
COF-367-Co^II/III^	Low-pressure solvothermal (50 mTorr). Schiff base condensation between Co^II/III^-TAPP (5,10,15,20-Tetrakis(4-aminophenyl)porphyrin and 4, 4′-biphenyldicarboxaldehyde.	Co^II^ and Co^III^ centered in porphyrin-based COF	[72]
Ru-TpPa-1	Anaerobic solvothermal Schiff base condensation between 1,3,5-triformylphloro-glucinol and p-phenylenediamine. RuCl_3_ added post-synthetically through incipient wetness, followed by reduction in H_2_/N_2._	Ketoamine-based COF loaded with Ru NPs enhancing the light absorption and charge separation	[73]
Z-Scheme SnS_2_/S-CTF	Anaerobic solvothermal nucleophilic substitution between trithiocyanuric acid and cyanuric chloride. SnS_2_ nanoparticles grown on framework through addition of SnCl_2_ and heating under autogenous pressure with EtOH.	Flower-like surface made of vertically aligned nanosheets in which SnS_2_ nanocrystals are attached with S-CTF framework	[74]
Re-TpBpy	Anaerobic solvothermal Schiff base condensation between 1,3,5-tri- formylphloroglucinol and 2,2′-bipyridine-5,5′-diamine. Post synthetic ligation of [Re(CO)_5_Cl].	Bipyridine units in COFs facilitating chelation with Re complex creating active sites on the porous walls	[75]
COF-367-Co Nanosheets	Solvothermal modulated imine exchange, between (5,10,15,20-Tetrakis(4-aminophenyl)porphyrin, 2,4,6-trimethylbenzaldehyde, and 4,4′-biphenyldialdehyde.	Ultrathin 2D imine-based Co-COF nanosheet structure with large surface area	[76]
DQTP- TMI	Solvothermal Schiff base condensation between 2,6-diaminoanthraquinone and 1,3,5-triformylphloroglucinol. Transition metal ions (TMIs) Co, Ni and Zn added post-synthetically via hydrothermal treatment.	2D anthraquinone based COF with conjugation and porosity assisting electron movement and CO_2_ adsorption	[77]

**Table 6:** COF-based hybrid materials for photocatalytic CO_2_ reduction.

COFs	Type	Light Source	Reaction Conditions	Photocatalytic Performance (μmol h^−1^g^−1^)	Ref.
COF-367-Co Nanosheets	Bipyridyl-imine-linked porphyrinic	300 W Xe (λ > 420 nm)	Catalyst (5 mg) 20 mL KOH _(aq)_ (0.1 M) 19 mg [Ru(bpy)_3_]Cl_2_.6H_2_O 352 mg ascorbic acid 1 atm CO_2,_ 25 °C	Nanosheet: 10,162 (CO) 2875 (H_2_) Bulk: 124 (CO)	[76]
Re-bpy-sp^2^c-COF	2D sp^2^c bipyridyl	300 W Xe (λ > 420 nm)	Catalyst (1 mg) 5 mL, 30:1 MeCN:TEOA 1 atm CO_2_	1040 (CO)	[11]
DQTP- TMI	2D anthraquinone	300 W Xe (λ > 420 nm)	Catalyst (20 mg) 50 mL, 4:1 MeCN:TEOA 22.5 mg Ru(bpy)_3_Cl_2_.H_2_O 1 atm CO_2,_ 25 °C	TMI = Co: 1022 (CO)	[77]
[Ni(bipy)_3_]^2+^—PI-COF	Covalent Triazine	300 W Xe (λ > 420 nm)	Catalyst (10 mg) Ni(ClO_4_)_2_.6H_2_O (2 mg) 2,2′-bipyridyl (15 mg) 5 mL, 3:1:1 MeCN: H_2_O:TEOA 1 atm CO_2_	483 (CO)	[70]
Re-TpBpy	2D bipyridyl-linked	200 W Xe (λ > 390 nm)	Catalyst (15 mg) 11.8 mL MeCN/H_2_O (10/1.8) 0.1 M TEOA 1 atm CO_2_, 25 °C Purpose-built reactor	291.7 (CO)	[75]
Z-Scheme SnS_2_/S-CTF	Covalent Triazine	300 W Xe (λ > 420 nm)	Catalyst (20 mg), 10 mL, 4:1 H_2_O:TEOA 1 atm CO_2_, 25 °C	123.6 (CO) 43.4 (CH_4_)	[74]
Ru-TpPa-1	Ketoamine-based	300 W Xe (λ > 420 nm)	Catalyst (15 mg) 110 mL, 10:1 MeCN:TEOA 1 atm CO_2_,	0 wt % Ru: 32.4 (HCOOH) 1 wt % Ru: 41.8 (HCOOH) 3 wt % Ru: 108.8 (HCOOH) 5 wt % Ru: 61.8 (HCOOH)	[73]
COF-367-Co^II/III^	Bipyridyl-imine-linked Porphyrinic	300 W Xe (λ > 380 nm)	Catalyst (10 mg) 22 mL, 10:1 MeCN:TEA 1 atm CO_2_	Co(II) 48.6 (HCOOH), 16.5 (CO), 12.8 (CH_4_) Co(III) 93.0 (HCOOH), 0.44 (CO), 10.1 (CH_4_)	[72]
Co-CTF-T1	Covalent Triazine	300 W Xe (λ > 420 nm)	Catalyst (10 mg) 7 mg [Ru(bpy)_3_]^2+^ 0.9 mL, 1:2:3 TEOA:H_2_O:MeCN 1 atm CO_2_	48 (CO)	[69]
N_3_-COF, ACOF-1	Covalent Triazine and Azine inked	500 W Xe (λ > 420 nm)	Catalyst (10 mg) 5 mL H_2_O 4 atm CO_2_, 80 °C	N_3_-COF: 0.57 (MeOH) ACOF-1: 0.36 (MeOH)	[71]

**Table 7:** Structure–activity correlations for enhancing the photocatalytic performance in CO_2_ reduction reaction.

COFs	Active Sites	Topology	Photocatalytic Performance (µmolh^−1^g^−1^)	Ref.
Co-CTF-1	COF and Ru(bpy)_3_^2+^: photosensitizer Co: redox active sites	2D-layered COF porous structure with triazine units promoting CO_2_ adsorption and accommodation	48 (CO)	[69]
[Ni(bipy)_3_]^2+^—PI-COF	COF: photosensitizer Ni: CO_2_ activation	Hexagonal porous polyimide COF assisting in CO_2_ photoreduction	483 (CO)	[70]
N_3_-COF, ACOF-1	COF: photosensitizer and redox active site	Coplanar azine and phenyl rings facilitating the conjugation effect	N_3_-COF: 0.57 (MeOH) ACOF-1: 0.36 (MeOH)	[71]
Re-bpy-sp^2^c-COF	COF: photosensitizer Re complex: electron mediator	Porous crystalline COF with bipyridine units ligated to Re complex, forming fully π-conjugated backbone	1040 (CO)	[11]
COF-367-Co^II/III^	COF: photosensitizer Co: redox active site	Co^II^ and Co^III^ centered in porphyrin-based COF	Co(II) 48.6 (HCOOH), 16.5 (CO), 12.8 (CH_4_) Co(III) 93.0 (HCOOH), 0.44 (CO), 10.1 (CH_4_)	[72]
Ru-TpPa-1	COF: photosensitizer Ru NPs: reductant	Ketoamine-based COF loaded with Ru NPs enhancing the light absorption and charge separation	0 wt % Ru: 32.4 (HCOOH) 1 wt % Ru: 41.8 (HCOOH) 3 wt % Ru: 108.8 (HCOOH) 5 wt % Ru: 61.8 (HCOOH)	[73]
Z-Scheme SnS_2_/S-CTF	SnS_2_ and COF: photosensitizer COF: CO_2_ adsorption and activation	Flower-like surface made of vertically aligned nanosheets in which SnS_2_ nanocrystals are attached with S-CTF framework	123.6 (CO) 43.4 (CH_4_)	[74]
Re-TpBpy	COF: photosensitizer Re complex: catalytic active site	Bipyridine units in COFs facilitating chelation with Re complex creating active sites on the porous walls	291.7 (CO)	[75]
COF-367-Co Nanosheets	Ru complex: photosensitizer Co-COF: active site	Ultrathin 2D imine-based Co-COF nanosheet structure with large surface area	Nanosheet: 10162 (CO) 2875 (H_2_) Bulk: 124 (CO)	[76]
DQTP- TMI	Ru complex: photosensitizer Co, Ni, Zn-COF: redox active site for CO_2_ adsorption and reduction	2D anthraquinone based COF with conjugation and porosity assisting electron movement and CO_2_ adsorption	TMI = Co: 1022 (CO)	[77]

**Table 8:** State-of-the-art photocatalysts for CO_2_ reduction.

Photocatalyst	Light Source	Reaction Conditions	Photocatalytic Performance (µmolh^−1^g^−1^)	Ref
Ag-Cr/Ga_2_O_3_	400 W Hg lamp (λ > 420 nm)	Catalyst (0.5 g), NaHCO_3_ (0.1 M), CO_2_ (30 mLmin^−1^)	480 (CO)	[85]
Cu@V-TiO_2_/PU	Two white light bulbs	CO_2_ (50 mLmin^−1^) passing through water at 303 K	933 (CH_4_)	[86]
ZnO nanosheets	125 W Hg lamp	Catalyst (50 mg), 0.5 mL deionized water at 473 K, CO_2_ (1 atm)	406.77 (CO) 20.16 (CH_4_)	[87]
Rh-Au-SrTiO_3_	500 W Xe lamp (λ > 400 nm)	Catalyst (75 mg), 3 mL deionized water and CO_2_ (70 kPa)	369 (CO)	[88]
Mo-doped g-C_3_N_4_	300 W Hg lamp	Catalyst (100 mg), 5.0 g deionized water at 303 K and CO_2_ (110 KPa)	887 (CO) 123 (CH_4_)	[89]
Nb-TiO_2_/g-C_3_N_4_	Two 30 W white bulbs	Catalyst (100 mg), CO_2_ (20 mL min^−1^) passing through water at 303 K	562 (CH_4_) 420 (CO) 698 (HCOOH)	[90]
